# Costs, compensations, and community building of implicit prosocial behavior in college dormitory environments: a mixed-methods study

**DOI:** 10.3389/fpsyg.2026.1922584

**Published:** 2026-10-19

**Authors:** Jingwei Li, TianYu Li, Pengcheng Li

**Affiliations:** 1 School of Engineering, Lishui University, Lishui, Zhejiang, China; 2 Faculty of Teacher Education, Lomonosov Moscow State University, Moscow, Russia; 3 College of Teacher Education, Lishui University, Lishui, Zhejiang, China

**Keywords:** compensation mechanism, implicit prosocial behavior, perceived cost, random intercept cross-lagged panel model, relational balance, sense of community, university dormitory

## Abstract

Implicit prosocial behaviors in university dormitories, such as daily assistance, emotional support, and accommodation of others' living habits, help maintain interpersonal relationships and community functioning, yet they may carry hidden costs that erode the willingness to continue. This mixed-methods project examined the cost structure, compensation mechanisms, and community-building processes of implicit prosocial behavior in dormitory contexts across four studies. Study 1 used grounded theory with 48 interviewed students to construct a “cost-compensation-community” model. Study 2 analyzed a two-wave longitudinal survey (*N* = 1856, six-month interval) with random intercept cross-lagged panel modeling. Study 3 tested cost intensity and compensation type in a 2 x 3 scenario experiment (*N* = 320). Study 4 evaluated a 10-week relationship-balance intervention in 54 dormitories (108 students) with a three-month follow-up. Implicit prosocial behavior comprised daily assistance, emotional support, and spatial coordination; four costs emerged (time, energy, emotional, boundary), with boundary cost carrying the largest relative weight (40.7%). Within persons, T1 cost perception negatively predicted T2 sustained willingness [standardized gamma = −0.37, 95% bootstrap CI (−0.42, −0.31)], whereas the reverse path was not significant. Relational balance statistically accounted for 62.0% of the concurrent cost-willingness association. Implicit relational rewards showed a stronger indirect association than explicit recognition, and dormitory sense of community buffered the cost-willingness link (interaction beta = 0.15). The experiment showed main effects of cost and compensation, with implicit rewards producing the highest willingness. The intervention improved balance, willingness, and community sense, with gains maintained at follow-up. Findings clarify the cost-compensation dynamics of quiet everyday helping and inform dormitory community practice.

## 1 Introduction

### 1.1 Kindness beneath the eaves: implicit prosocial behavior in dormitory environments

The university dormitory is a distinctive micro-social space in which four to six students share a few square meters and live together day and night. Within such shared quarters, a large amount of interpersonal care takes place quietly: taking out the trash without being asked, bringing meals to a sick roommate, listening to a roommate's worries late at night, and adjusting one's own routines to accommodate others. These acts are not grand heroic deeds; they are small, recurrent forms of everyday helping whose value accumulates over time. Recent evidence indicates that such everyday acts of kindness are meaningfully linked to the wellbeing of students during the transition to university (Cash et al., 2024), and meta-analytic work confirms a reliable, bidirectional-looking association between prosociality and wellbeing (Hui et al., 2020; Ju et al., 2025).

We define implicit prosocial behavior as voluntary actions in daily interaction that intend to benefit others or the shared living environment, are performed habitually and without deliberation, and are neither formally recorded nor deliberately made visible to others. Three boundary conditions separate this construct from its nearest neighbors. First, unlike formal prosocial behavior studied in most of the literature, such as volunteering, donation, or organized helping (Penner et al., 2005), implicit prosocial behavior is informal, low in visibility, and embedded in ongoing relationships rather than in structured roles. Second, it is related to but conceptually distinct from everyday prosocial behavior as used in daily-diary research: everyday helping is defined mainly by frequency and ordinariness, whereas the present construct also requires low expressiveness, that is, the act is typically not announced, acknowledged, or socially documented. Third, it differs from altruistic motivation: the construct describes observable behavior rather than a motivational state, and, as we show below, its sustainability depends heavily on perceived returns. In the dormitory context, implicit prosocial behavior shows five features: daily occurrence, small individual magnitude with cumulative impact, spontaneity and habitual execution, low visibility, and embeddedness in long-term reciprocal relationships (Clark and Mills, 1979; McMillan and Chavis, 1986).

### 1.2 The cost of goodwill: the cost structure of implicit prosocial behavior

Goodwill is not free. Even small acts of helping consume resources, and in the confined dormitory environment these costs are particularly sensitive because exit options are limited and interaction is unavoidable. Drawing on resource and exchange perspectives (Cropanzano and Mitchell, 2005), we distinguish four costs. Time cost arises when helping roommates consumes time that could be used for study, entertainment, or rest. Energy cost follows from the attentional and physical effort of dealing with roommates‘ needs, scheduling conflicts, and dormitory chores. Emotional cost appears when unseen dedication is taken for granted and the balance between giving and receiving feels persistently uneven, which can breed disappointment or resentment. Boundary cost, finally, is the most context-specific: when individuals habitually accommodate others, personal space, privacy, and control over one's own schedule are progressively given up. Privacy regulation and territorial control are basic needs in shared environments (Altman, 1975), and negotiating disclosure and privacy is a recurring task in close relationships (Kil et al., 2022); in a confined space with already fragile boundaries, accumulated implicit helping can further weaken individuals' sense of control over personal space, time, and privacy. Research on the costs of prosocial behavior has concentrated on tangible costs such as money or time, and has paid less attention to emotional and boundary costs of informal, low-visibility helping; boundary cost may therefore be a key factor for the sustainability of implicit prosocial behavior in dormitories.

### 1.3 Cost compensation: the sustainability conditions of implicit prosocial behavior

If implicit prosocial behavior is costly, what sustains it? Social exchange theory offers the baseline account: behavior persists when perceived rewards exceed perceived costs (Cropanzano and Mitchell, 2005). In dormitories, rewards take two forms. Explicit compensation consists of directly expressed gratitude, verbal acknowledgment, and tangible reciprocation, which matches the classical exchange template. Implicit compensation consists of rising relational warmth, accumulated trust, a sense of being needed, and a feeling of belonging, which fits communal rather than strictly exchanged-based relationships (Clark and Mills, 1979). Gratitude research shows that expressed appreciation functions relationally by finding, reminding, and binding partners, which suggests that even implicit rewards may operate through relationship perception rather than material payoff (Emmons and McCullough, 2003). Between costs and compensation stands the sense of relational balance, the individual's perception that effort and reward are roughly equal over time; we treat this balance perception as the central psychological mechanism that translates costs and compensations into the decision to continue or withdraw.

### 1.4 From individual goodwill to community building

Implicit prosocial behavior is not only an individual-level expression of goodwill but also a micro-foundation of community building. When such behaviors circulate reciprocally among dormitory members, a psychological sense of community, with membership, influence, fulfillment of needs, and shared emotional connection as its elements, can emerge and stabilize (McMillan and Chavis, 1986). University students' sense of community is reliably associated with their wellbeing (Zarbo et al., 2025), and roommate relationships behind closed doors are a consequential but understudied part of residential life (Yang, 2025). A community, however, depends on a dynamic balance between costs and compensations: when costs accumulate while compensation lags, members may disengage, trust thins, and the reciprocal cycle stalls. Understanding this dynamic has practical value for strengthening cohesive dormitory communities.

### 1.5 Research framework and questions

Bringing these strands together, this project builds an integrated “cost-compensation-community” framework (Figure 1) and examines it in four studies: (1) What are the typical manifestations of implicit prosocial behavior in university dormitories? (2) What costs does it entail, and how does the relative weight of each cost dimension compare? (3) How does cost perception influence the sustained willingness for implicit prosocial behavior over time, and is relational balance a mechanism of this association? (4) How does the compensation mechanism operate, and does implicit compensation matter more than explicit compensation? (5) Does dormitory sense of community moderate the association between cost and sustained willingness? (6) Is a reciprocity-based intervention program effective in improving balance, willingness, and community sense?

**Figure 1:**
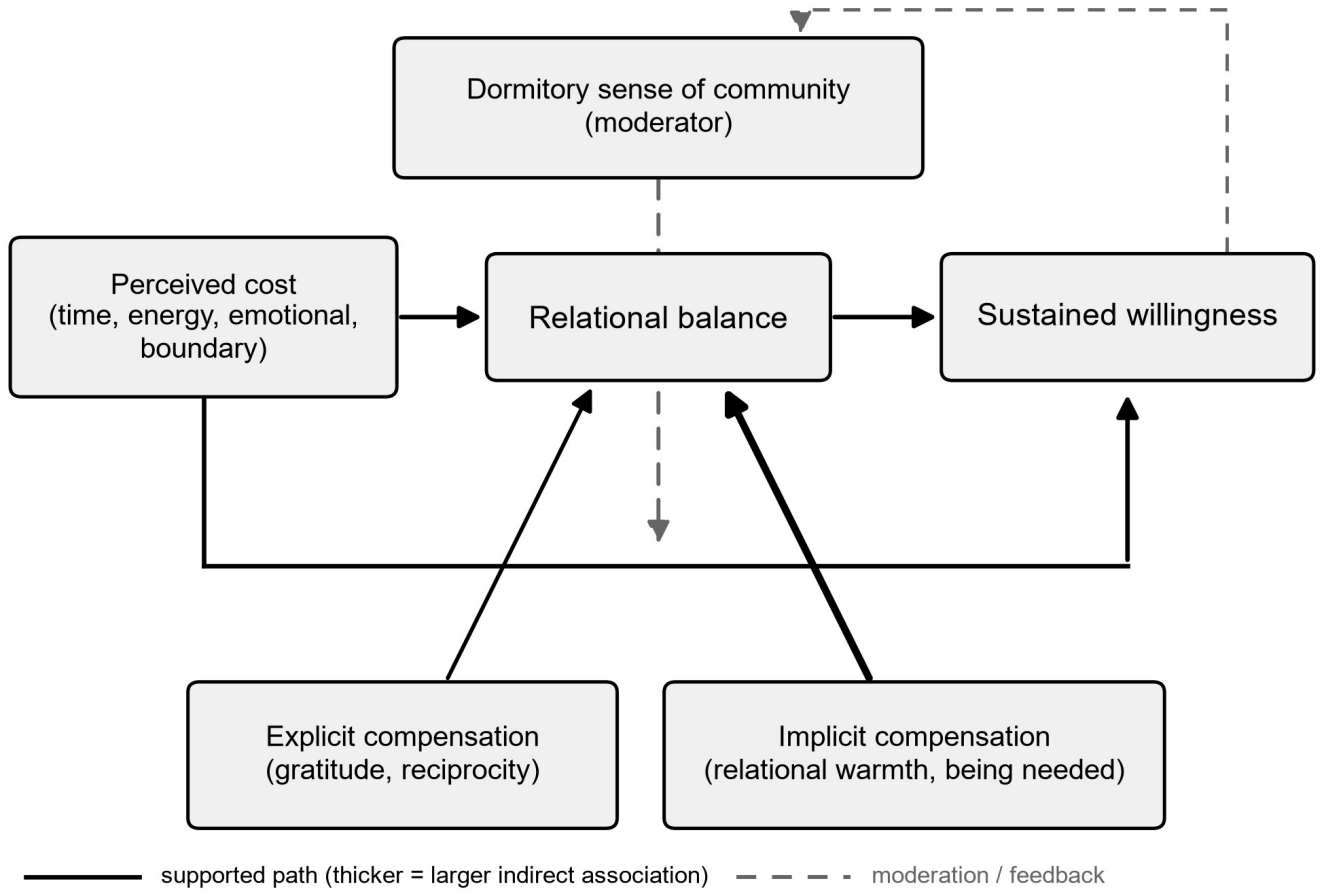
The cost-compensation-community framework of implicit prosocial behavior in university dormitories. Perceived costs (time, energy, emotional, and boundary) undermine relational balance and, through it, sustained willingness to help; explicit and implicit compensations feed back into balance perception, with implicit rewards carrying the larger indirect association. Solid arrows mark the paths supported in Studies 2–4 (line weight indicates the relative magnitude of the indirect associations); dashed arrows mark the moderation of the cost-willingness path by dormitory sense of community and the feedback from sustained helping into community building.

## 2 Study 1: grounded theory of implicit prosocial behavior in dormitories

### 2.1 Aim

Study 1 used grounded theory to examine the conceptual structure, cost characteristics, and compensation mechanisms of implicit prosocial behavior in university dormitory settings, and to construct a theoretical model that could be tested quantitatively in Studies 2–4 (Strauss and Corbin, 1998).

### 2.2 Participants

Using theoretical sampling, 48 college students with dormitory living experience were recruited (20 men, 28 women; 8 first-year, 12 second-year, 14 third-year, 10 fourth-year, and 4 postgraduate students; 4-person and 6-person rooms). Inclusion criteria were: (1) at least one year of dormitory living experience; (2) self-reported frequent informal helping of roommates; and (3) willingness to reflect on dormitory relationships. Criterion 2 deliberately sampled frequent helpers to ensure rich accounts; the resulting self-selection is addressed as a limitation.

### 2.3 Data collection

Semi-structured in-depth interviews covered five topics: daily acts of mutual assistance in the dormitory; feelings and thoughts when helping roommates; motivations and difficulties of continued helping; perceived balance between effort and reward; and changes in dormitory relationships and sense of community. Interviews lasted 60–120 min, were audio recorded with consent, and were transcribed verbatim, yielding approximately 550,000 Chinese characters of text. Sampling stopped when two consecutive interviews produced no new conceptual codes, indicating theoretical saturation.

### 2.4 Data analysis

Analysis followed the three-stage coding procedure of Strauss and Corbin (open, axial, and selective coding) supported by NVivo 14 (Strauss and Corbin, 1998). Two trained coders independently coded all transcripts; initial between-coder agreement at the category level was 84.7%, and all disagreements were resolved through discussion with a third member of the research team until full consensus was reached. An audit trail of memos documented coding decisions and category revisions.

### 2.5 Findings

#### 2.5.1 Open and axial coding

Line-by-line coding produced 326 initial concepts that were merged into 27 initial categories and then into 12 main categories. Illustrative examples: “Every day it is me who takes out the trash; my roommates seem to have gotten used to it” (daily assistance); “During my roommate's heartbreak I stayed up chatting with her every night” (emotional support); “My roommate sleeps early, so I move quietly and mute my phone” (spatial coordination); “Sometimes I am really tired but still bring them food” (time cost); “After hearing them complain again and again, I became affected by the negative mood” (emotional cost); “I feel my personal space is shrinking; even deciding when to sleep requires considering others” (boundary cost); “My roommate said thank you, and I felt it was worth it” (explicit compensation); “We are growing closer, like family” (implicit compensation); “What I give is roughly balanced with what I receive” (relational balance); “Lately I feel less inclined to get involved; no one cares anyway” (withdrawal after imbalance).

#### 2.5.2 Selective coding and the theoretical model

Selective coding identified the core category as the “cost-compensation-balance mechanism of implicit prosocial behavior.” The storyline is as follows. Within dormitories, implicit prosocial behavior takes three forms: daily assistance, emotional support, and spatial coordination. These behaviors entail four costs (time, energy, emotional, boundary), among which boundary cost is the most contextually specific and, as Study 2 shows, the most consequential. Cost perception relates to sustained willingness through relational balance: when giving and receiving feel roughly balanced, willingness stays high; prolonged imbalance invites withdrawal. The compensation system comprises explicit rewards and implicit rewards, which jointly inform balance perception. Dormitory sense of community (belonging, trust, and reciprocity norms) conditions how strongly costs translate into withdrawal, and community building feeds back into further giving. This model guided the measurement and hypotheses of Studies 2–4 (Figure 1).

## 3 Study 2: longitudinal mechanisms of cost, balance, and willingness

### 3.1 Aim

Study 2 tested the theoretical model from Study 1 in a large two-wave longitudinal survey, examining how cost perception relates to sustained willingness over time, the concurrent mechanism of relational balance, the indirect role of compensation perceptions, and the moderating role of dormitory sense of community.

### 3.2 Participants and procedure

Using multi-stage cluster sampling, questionnaires were distributed to students of 12 universities in the eastern, central, and western regions of China within a longitudinal project running from March 2025 to March 2026; Wave 1 (T1) took place in March 2025 and Wave 2 (T2) in September 2025, a six-month interval. At T1, 2,500 questionnaires were distributed and 2,108 valid responses were returned (valid rate 84.3%); 1856 students were successfully matched at T2 (retention 88.0%). The matched sample comprised 620 men (33.4%) and 1236 women (66.6%), aged 18–26 years (*M* = 20.37, SD = 1.52); 68.5% lived in 4-person rooms, 24.3% in 6-person rooms, and 7.2% in other room types. Participants completed the survey online in class time, gave informed consent, and were assured of confidentiality; gender, age, grade, room type, and length of residence were recorded as covariates.

### 3.3 Measures

All measures were developed from the grounded categories of Study 1 and used 5-point Likert response scales (1 = never or strongly disagree, 5 = always or strongly agree); the full item pool is provided in Supplementary Table S1. The Implicit Prosocial Behavior Scale (15 items) covers daily assistance (five items, e.g., “I proactively take out the dormitory trash”), emotional support (five items, e.g., “I listen to my roommates‘ worries”), and spatial coordination (five items, e.g., “I adjust my routine to my roommates' schedules”); alpha = 0.91. The Cost Perception Scale (13 items) covers time (three items), energy (three items), emotional (three items), and boundary costs (four items, e.g., “My personal space feels eroded”); alpha = 0.90. The Compensation Perception Scale (eight items) distinguishes explicit rewards (four items, e.g., “My roommates express clear gratitude”) from implicit rewards (four items, e.g., “We are becoming closer”); alpha = 0.88. The Relational Balance Scale (six items, e.g., “My effort and reward feel balanced”) had alpha = 0.87. The Sustained Willingness Scale (five items, e.g., “I am willing to keep helping my roommates”) had alpha = 0.89. The Dormitory Sense of Community Scale (nine items) covers belonging, trust, and reciprocity norms (three items each); alpha = 0.89. All six scales were administered at T1; cost perception, compensation perception, relational balance, and sustained willingness were re-administered at T2.

### 3.4 Analytic strategy

Analyses were conducted in Python 3.13 [Python Software Foundation, Beaverton, OR, United States (https://www.python.org)] using semopy 2.3.11 and statsmodels 0.15. Common method bias was screened with Harman's single-factor test (Podsakoff et al., 2003, 2024). The retained sample was randomly split in half: exploratory factor analysis (principal axis factoring with varimax rotation) was run in the first half (*n* = 928), and confirmatory factor analysis in the second half (*n* = 928), evaluating CFI, TLI, RMSEA, and SRMR against conventional cutoffs (Hu and Bentler, 1999), with omega as the reliability estimate (Dunn et al., 2014) and HTMT ratios for discriminant validity (Henseler et al., 2015). Longitudinal measurement invariance across waves was tested at the configural, metric, and scalar levels, allowing residuals of matched indicators to covary across waves as is standard in longitudinal models. Reciprocal longitudinal associations between cost perception and sustained willingness were estimated with a random intercept cross-lagged panel model (RI-CLPM) (Hamaker et al., 2015; Mulder and Hamaker, 2021), using four cost parcels and the five willingness items as indicators, with loadings and residual variances constrained equal across waves for identification; inference for the cross-lagged paths used 500 bootstrap resamples. A single-indicator sensitivity analysis with reliabilities fixed at 0.70, 0.80, and 0.90 checked the stability of the cross-lagged estimates (Mulder and Hamaker, 2021), and a classical CLPM was fitted for comparison. Indirect associations at T1 were estimated with 5,000 bias-corrected bootstrap resamples (Preacher and Hayes, 2008; Wen and Ye, 2014) and are interpreted as concurrent decompositions rather than longitudinal mediation (Maxwell and Cole, 2007). Moderation was tested with hierarchical regression on mean-centered scores, and the relative importance of the four cost dimensions was compared with dominance analysis (Budescu, 1993; Mizumoto, 2022).

### 3.5 Results

#### 3.5.1 Common method bias

Harman's single-factor test showed that the first unrotated factor accounted for 27.5% of the total variance, below the 40% reference value, suggesting that common method bias is unlikely to account for the observed associations (Podsakoff et al., 2003).

#### 3.5.2 Descriptive statistics and correlations

Means, standard deviations, reliabilities, and correlations of the T1 study variables are shown in Table 1. Cost perception correlated negatively with compensation perception, relational balance, sustained willingness, and sense of community (*r* = −0.48 to −0.42), whereas compensation, balance, willingness, and community correlated positively with each other (all *p* < 0.001).

**Table 1:** Means, standard deviations, internal consistencies, and correlations of the Study 2 variables (*N* = 1856).

Variable	*M*	SD	Alpha	1	2	3	4	5
1. Perceived cost	2.85	0.72	0.90	-	-	-	-	-
2. Compensation	3.52	0.68	0.88	−0.48	-	-	-	-
3. Relational balance	3.45	0.74	0.87	−0.56	0.62	-	-	-
4. Sustained willingness	3.62	0.71	0.89	−0.48	0.55	0.63	-	-
5. Sense of community	3.55	0.69	0.89	−0.42	0.58	0.59	0.58	-
6. Implicit prosocial behavior	3.88	0.60	0.91	-	-	-	-	-

Variables 1–5 are T1 composite scores; all reported correlations are significant at *p* < 0.001. Implicit prosocial behavior (IPB) is shown for descriptive reference. The sample comprised 620 men and 1236 women (age *M* = 20.37, SD = 1.52); room types were 68.5% four-person, 24.3% six-person, and 7.2% other. Of 2,500 distributed questionnaires, 2,108 were valid at T1 (84.3%) and 1856 were tracked at T2 (88.0%).

#### 3.5.3 Psychometric evaluation of the self-developed scales

In the exploratory half, the intended factor structures were recovered for all six scales: eigenvalue-greater-than-one criteria matched the designed dimension numbers (3, 4, 2, 1, 1, and 3 factors respectively), KMO values ranged from 0.88 to 0.95, Bartlett tests were all significant (*p* < 0.001), primary loadings were at least 0.55, and cross loadings did not exceed 0.34 (full loading matrices in Supplementary Table S2). In the confirmatory half, all six models fit well: CFI ≥ 0.998, TLI ≥ 0.997, RMSEA < = 0.020, and SRMR < = 0.018 (Table 2). Omega coefficients ranged from 0.93 to 0.96, average variance extracted from 0.49 to 0.76 (one spatial-coordination factor marginally below 0.50), and composite reliabilities from 0.83 to 0.91. Discriminant validity was supported: the HTMT ratio between boundary cost and emotional cost, the two conceptually closest cost dimensions, was 0.48, well below the 0.85 criterion (Henseler et al., 2015), and all remaining HTMT ratios were below 0.64.

**Table 2:** Split-half psychometric evaluation of the six measures.

Measure	Items	KMO	Var. %	CFI	TLI	RMSEA	SRMR	Omega	AVE
Implicit prosocial behavior	15	0.950	61.9	0.998	0.998	0.012	0.018	0.954	0.49–0.51
Perceived cost	13	0.880	81.5	1.000	1.000	0.006	0.015	0.958	0.71–0.76
Compensation	8	0.889	73.1	1.000	1.003	0.000	0.009	0.942	0.60–0.63
Relational balance	6	0.907	61.8	0.998	0.997	0.020	0.012	0.928	0.50–0.50
Sustained willingness	5	0.887	69.4	1.000	1.000	0.000	0.007	0.946	0.61–0.61
Sense of community	9	0.892	75.9	1.000	1.000	0.004	0.013	0.940	0.63–0.64

Exploratory factor analysis on a random half of the sample (KMO, percentage of variance explained by the retained factors; Bartlett tests were significant for all measures) and confirmatory factor analysis on the holdout half. AVE = average variance extracted (range across subdimensions); Omega = McDonald's omega total. Full item lists and loading matrices are in Supplementary Tables S1, S2.

#### 3.5.4 Longitudinal measurement invariance

For the four constructs measured at both waves, configural, metric, and scalar models all fit the data well (all CFI > 0.99, RMSEA < 0.01), and the change in CFI between successive constraints did not exceed 0.001 (delta RMSEA < = 0.001), supporting scalar invariance over the six-month interval; latent means and longitudinal paths can therefore be compared across waves.

#### 3.5.5 Cost perception and sustained willingness: random intercept cross-lagged panel model

The RI-CLPM separated stable between-person differences (random intercepts) from within-person fluctuations and fit the data well: chi-square(134) = 146.22, *p* = 0.222, CFI = 0.999, TLI = 0.999, RMSEA = 0.007, SRMR = 0.020 (Table 3). Within persons, T1 cost perception negatively predicted T2 sustained willingness [gamma = −0.44, standardized −0.37, 95% bootstrap CI (−0.42, −0.31)], whereas T1 willingness did not predict T2 cost perception [gamma = 0.02, standardized 0.02, 95% bootstrap CI (−0.14, 0.16)]. Autoregressive within-person carry-over was moderate for both constructs (cost phi = 0.43, willingness phi = 0.48), and the random intercepts covaried negatively (*r* = −0.87), indicating that students with chronically higher cost perception reported chronically lower willingness. The single-indicator sensitivity analysis reproduced the asymmetric pattern across reliability assumptions (standardized gamma12 = −0.38 to −0.38; gamma21 = −0.04 to 0.15). A classical CLPM yielded a weaker cost-to-willingness path (−0.29) and a small reverse path (−0.11), illustrating how failing to separate trait variance attenuates and blurs within-person estimates (Alameer et al., 2026; Hamaker et al., 2015). Because only two waves were available, these within-person estimates describe short-run directional influence rather than an identified causal chain, and are interpreted accordingly.

**Table 3:** Random intercept cross-lagged panel model estimates.

Parameter	*b*	Beta	95% CI
Cost T1 to willingness T2 (cross-lagged)	−0.44	−0.37	[−0.417, −0.309]
Willingness T1 to cost T2 (cross-lagged)	0.02	0.02	[−0.139, 0.162]
Cost autoregression (T1 to T2)	0.43	-	-
Willingness autoregression (T1 to T2)	0.48	-	-
Random intercept correlation	−0.87	-	-
Classical CLPM: cost T1 to willingness T2	−0.29	-	-
Classical CLPM: willingness T1 to cost T2	−0.11	-	-

Model fit: chi-square(134) = 146.22, *p* = 0.222, CFI = 0.999, TLI = 0.999, RMSEA = 0.007, SRMR = 0.020. Cross-lagged inferences rely on 500-draw bootstrap confidence intervals of the standardized estimates because numerical Hessian standard errors were unstable under the flat ridge between random-intercept variances and autoregressive paths. Sensitivity analyses assuming scale reliabilities of 0.70, 0.80, and 0.90 yielded standardized cost-to-willingness estimates of −0.380, −0.404, −0.380, respectively. Classical cross-lagged panel model estimates are shown for comparison.

#### 3.5.6 Relational balance as a concurrent mechanism

At T1, the association between cost perception and sustained willingness decomposed into a direct path [*b* = −0.18, 95% CI (−0.22, −0.14)] and an indirect path through relational balance [*b* = −0.29, 95% CI (−0.32, −0.26); standardized −0.30], with the indirect path accounting for 62.0% of the total association (Table 4). Because this decomposition is cross-sectional, the percentage should be read as a description of the associative structure rather than as evidence of a temporal mediating process (Maxwell and Cole, 2007); it is consistent with, but does not by itself establish, the balancing mechanism proposed in Study 1.

**Table 4:** Cross-sectional decomposition of the cost-willingness association through relational balance (T2).

Effect	*b*	95% CI	Beta	95% CI
Indirect via relational balance	−0.292	[−0.322, −0.263]	−0.297	[−0.324, −0.271]
Direct	−0.179	[−0.220, −0.137]	−0.182	[−0.222, −0.141]
Total	−0.471	[−0.513, −0.430]	−0.479	[−0.513, −0.442]
Proportion mediated, %	62.0	-	62.0	-

Confidence intervals derive from 5,000 bias-corrected bootstrap resamples. Because the decomposition is cross-sectional, the proportion mediated is reported for descriptive purposes only and should not be read as evidence of a temporal indirect pathway.

#### 3.5.7 Compensation perceptions and relational balance

A parallel indirect-effects model linked cost perception to relational balance through the two compensation perceptions (Table 5). The indirect association through implicit rewards [standardized −0.20, 95% CI (−0.23, −0.18)] was significantly larger than that through explicit rewards [standardized −0.04, 95% CI (−0.06, −0.03)]; the difference between the two was itself significant [contrast = 0.16, 95% CI (0.13, 0.19)]. In terms of the total cost-balance association, the two indirect paths corresponded to 36.4% (implicit) and 8.0% (explicit), respectively, indicating that relational warmth and being needed play the larger role in preserving balance perception.

**Table 5:** Indirect effects of cost perception on willingness through explicit and implicit compensation (T3).

Effect	*b*	95% CI	Beta	95% CI
Indirect via explicit compensation	−0.045	[−0.061, −0.031]	−0.044	[−0.060, −0.030]
Indirect via implicit compensation	−0.208	[−0.235, −0.181]	−0.203	[−0.228, −0.178]
Contrast (implicit minus explicit)	0.162	[0.130, 0.197]	0.159	[0.125, 0.190]
Direct effect	–.318	-	-	-

Confidence intervals derive from 5,000 bias-corrected bootstrap resamples. The explicit and implicit pathways accounted for 8.0% and 36.4% of the total standardized association, respectively; T3 measures were collected concurrently, so these estimates describe concurrent indirect associations.

#### 3.5.8 Dormitory sense of community as a moderator

A hierarchical regression on mean-centered scores showed that the negative association between cost perception and sustained willingness was qualified by dormitory sense of community (interaction *b* = 0.15, SE = 0.02, *t* = 9.02, *p* < 0.001; delta *R*^2^ = 0.025; Table 6). Simple slope estimates indicated that the cost-willingness slope was steeper at low community sense (-1 SD: *b* = −0.43, SE = 0.03, *p* < 0.001) than at high community sense (+1 SD: *b* = −0.13, SE = 0.03, *p* < 0.001), consistent with a buffering account.

**Table 6:** Hierarchical regression of sustained willingness on perceived cost, sense of community, and their interaction.

Predictor	*b*	SE	*t*	*p*
Perceived cost (centered)	−0.28	0.02	−14.52	< 0.001
Sense of community (centered)	0.45	0.02	23.10	< 0.001
Cost x community	0.15	0.02	9.02	< 0.001
Simple slope at low community (−1 SD)	−0.43	0.03	−17.14	< 0.001
Simple slope at high community (+1 SD)	−0.13	0.03	−5.10	< 0.001

Predictors were mean-centered before computing the product term. R2 = 0.402 for the main-effects model and R2 = 0.427 for the full model (delta R2 = 0.025).

#### 3.5.9 Relative importance of the four cost dimensions

Dominance analysis, which partitions the explained variance by averaging each predictor's incremental contribution over all subset models (Budescu, 1993; Yin and Zhou, 2025), showed that boundary cost carried the largest share of the explained variance in sustained willingness (40.7%), followed by emotional cost (30.0%), energy cost (17.6%), and time cost (11.7%; Table 7). The dominance ordering was complete and matched the grounded-theory account in which erosion of personal boundaries is the most consequential cost in confined shared housing.

**Table 7:** Dominance analysis of the four cost dimensions predicting sustained willingness.

Cost dimension	Relative weight	% of explained variance
Time cost	0.028	11.7
Energy cost	0.042	17.6
Emotional cost	0.072	30.0
Boundary cost	0.098	40.7

Relative weights average each predictor's incremental contribution over all subset models (total R2 = 0.240). The dominance ordering was complete: boundary > emotional > energy > time.

## 4 Study 3: scenario experiment on cost intensity and compensation type

### 4.1 Aim and design

Study 3 tested the causal logic implied by Studies 1–2 with a 2 (cost intensity: high vs. low) x 3 (compensation type: explicit reward, implicit reward, none) between-subjects scenario experiment. The primary outcome was sustained willingness; relational balance served as a secondary outcome, and cost and compensation perceptions as manipulation checks. An *a priori* power analysis (G^*^Power 3.1; *f* = 0.25, alpha = 0.05, power = 0.80, numerator df = 2, six groups) required at least 211 participants for a medium-sized effect; we recruited 320, giving power of 0.80 to detect interaction effects of *f* ≥ 0.17.

### 4.2 Participants and randomization

A total of 320 undergraduates (64.1% women; mean age 20.3 years, SD = 1.45) were recruited from one university and randomly assigned to the six conditions (ns = 52–54 per cell) using a computer-generated random sequence prepared in advance by a researcher not involved in data collection; allocation was concealed from participants, who were unaware of the study hypotheses.

### 4.3 Materials and procedure

Scenario materials depicted a protagonist who had long helped roommates (bringing meals, listening, accommodating routines), with cost intensity and compensation type varied across versions. High-cost scenarios described substantial time investment, academic interference, and restricted personal space; low-cost scenarios described minor time use and little interference. In the explicit-reward version, roommates expressed clear gratitude, reciprocated favors, and gave a small gift; in the implicit-reward version, the relationship grew warmer, the atmosphere more family-like, and the protagonist felt needed; in the no-compensation version, roommates took the help for granted. Materials were piloted with 60 students (not in the main sample), which confirmed scenario comprehensibility and the intended manipulation contrast. After reading, participants rated cost perception, compensation perception, relational balance, and sustained willingness.

### 4.4 Results

#### 4.4.1 Manipulation checks

Cost perception was higher in the high-cost than in the low-cost conditions [*M* = 3.90, SD = 0.80 vs. *M* = 3.36, SD = 0.80; F(1, 318) = 36.45, *p* < 0.001], and compensation perception differed across compensation conditions [F(2, 317) = 29.20, *p* < 0.001], with the no-compensation condition lowest (*M* = 3.10). Neither manipulation leaked into the unintended check [cost manipulation on compensation perception: *F* = 0.01, *p* = 0.913; compensation manipulation on cost perception: *F* = 0.01, *p* = 0.987].

#### 4.4.2 Effects on sustained willingness and relational balance

A 2 x 3 between-subjects ANOVA on sustained willingness (Table 8) showed significant main effects of cost intensity [F(1, 314) = 23.53, *p* < 0.001, partial eta2 = 0.070] and of compensation type [F(2, 314) = 51.63, *p* < 0.001, partial eta2 = 0.247]: willingness was lower under high cost, and highest under implicit rewards, followed by explicit rewards, with the no-compensation condition lowest. The interaction was not significant [F(2, 314) = 0.65, *p* = 0.524, partial eta2 = 0.004]. The same pattern held for relational balance [compensation main effect F(2, 314) = 11.59, *p* < 0.001, partial eta2 = 0.069; cost main effect and interaction ns].

**Table 8:** Two-way ANOVA of cost intensity and compensation type on sustained willingness and relational balance (Study 3, *N* = 320).

Effect	df	*F*	*p*	Partial eta2
Willingness: cost intensity	1	23.53	< 0.001	0.070
Willingness: compensation type	2	51.63	< 0.001	0.247
Willingness: cost x compensation	2	0.65	0.524	0.004
Balance: cost intensity	1	1.73	0.190	0.005
Balance: compensation type	2	11.59	< 0.001	0.069
Balance: cost x compensation	2	0.18	0.835	0.001

Cell statistics - high cost, explicit compensation: willingness 3.52 (0.68), balance 3.55 (0.70), repair rate 23.5%; high cost, implicit compensation: willingness 3.85 (0.65), balance 3.68 (0.70), repair rate 35.1%; high cost, no compensation: willingness 2.85 (0.72), balance 3.18 (0.70), repair rate reference; low cost, explicit compensation: willingness 3.85 (0.62), balance 3.62 (0.70), repair rate 16.0%; low cost, implicit compensation: willingness 4.12 (0.58), balance 3.75 (0.70), repair rate 24.1%; low cost, no compensation: willingness 3.32 (0.68), balance 3.35 (0.70), repair rate reference. Error df = 314 for all tests. Planned contrasts showed implicit compensation exceeding explicit compensation under high cost [t(105) = 2.57, *p* = 0.012, *d* = 0.50] and under low cost [t(106) = 2.34, *p* = 0.021, *d* = 0.45]; both compensation types exceeded the no-compensation condition (all *p* < 0.001). Manipulation checks confirmed the intended differences [cost perception: F(1, 318) = 36.45, *p* < 0.001; compensation perception: F(2, 317) = 29.20, *p* < 0.001] with no crossover effect (*F* = 0.01, *p* = 0.987). Observed power of the willingness interaction was 0.16; the non-significant interaction is therefore interpreted cautiously.

#### 4.4.3 Planned contrasts and compensatory repair

Planned contrasts showed that implicit rewards produced higher willingness than explicit rewards both under high cost (*t* = 2.57, *p* = 0.012, *d* = 0.50) and under low cost (*t* = 2.34, *p* = 0.021, *d* = 0.45), and both reward types exceeded the no-compensation condition (all *p* < 0.001). Simple effects of compensation type were significant within each cost level [high: F(2, 157) = 29.43; low: F(2, 157) = 22.22, both *p* < 0.001]. As a descriptive repair index, willingness gains relative to the no-compensation condition were 35.1% (implicit) and 23.5% (explicit) under high cost, and 24.1% and 16.0% under low cost. The repair advantage of implicit over explicit rewards was thus visible at both cost levels and descriptively larger under high cost, although the corresponding interaction did not reach significance, so the high-cost amplification should be treated as suggestive rather than established.

## 5 Study 4: a dormitory-clustered “relationship balance” intervention

### 5.1 Aim

Study 4 translated the mechanism findings into a 10-week group intervention built on reciprocity norms and evaluated it with a dormitory-clustered pre-post-follow-up design.

### 5.2 Participants and cluster randomization

Participants were 108 undergraduates from 54 dormitories who reported elevated cost perception and low relational balance. From each dormitory, two members (the focal pair) enrolled in the study; intervention exercises were practiced within the pair and, where feasible, extended to the remaining roommates. The dormitory, not the individual, was the unit of randomization: the 54 dormitories were randomly assigned to the intervention (27 dormitories, 54 students) or a waiting-list control condition (27 dormitories, 54 students) using a computer-generated sequence. Baseline comparisons showed no significant between-condition differences on any study variable (*t* = −0.23 to −0.24, all *p* > 0.75). Measurements took place before the intervention (T0), immediately after (T1), and three months later (T2).

### 5.3 Intervention protocol and implementation

The “relationship balance” program comprised five phases delivered in ten weekly 90-min sessions at the dormitory level: cost expression training (weeks 1–2; recognizing and voicing one's own contributions and costs using nonviolent communication); gratitude habit cultivation (weeks 3–4; weekly gratitude sharing with specific rather than generic thanks); reciprocal ritual building (weeks 5–6; jointly agreed accommodation rules and recurring reciprocal ceremonies); boundary negotiation skills (weeks 7–8; articulating personal boundaries and negotiating schedules and space); and community building with a self-sustaining mechanism (weeks 9–10; reviewing change, setting a shared vision, and scheduling monthly dormitory meetings). Sessions were co-led by two trained counselors following a session-by-session manual; mean attendance was 9.3 of 10 sessions, and fidelity checklists completed after each session indicated full delivery of all planned components. Control dormitories received no program during the study and were offered the program after follow-up.

### 5.4 Analytic strategy

Because students were nested in dormitories, intraclass correlations were computed for every outcome and wave, and linear mixed models with a random dormitory intercept (restricted maximum likelihood) served as the primary analysis, with fixed effects of time, condition, and their interaction; the interaction terms are difference-in-differences estimates of the intervention effect at post-test and follow-up. Mixed repeated-measures ANOVAs are reported as a sensitivity analysis, and standardized effects as between-condition Cohen's d at T1 and T2 and within-intervention change scores. The mechanism hypothesis from Studies 1–3, that compensation gains restore balance which in turn sustains willingness, was tested with a sequential indirect-effects model on T0-to-T1 change scores using 5,000 dormitory-cluster bootstrap resamples (resampling whole dormitories).

### 5.5 Results

#### 5.5.1 Intraclass correlations and intervention effects

Dormitory-level ICCs ranged from 0.11 to 0.51 across outcomes and waves, confirming the need for cluster-aware modeling. Mixed-model estimates (Table 9) showed significant time-by-condition interactions for all five outcomes: cost perception decreased in the intervention condition relative to control (T1: *b* = −0.56, SE = 0.15, *p* < 0.001; T2: *b* = −0.51, *p* < 0.001), whereas compensation perception, relational balance, sustained willingness, and sense of community increased (T1 interaction *b* = 0.64 to 0.75; T2 interaction *b* = 0.61 to 0.70; all *p* < 0.001). Between-condition effects were medium to large at post-test (*d* = 0.88 to 1.10) and remained substantial at the three-month follow-up (*d* = 0.76 to 0.98). Mixed repeated-measures ANOVAs produced convergent time-by-condition interactions [F(2, 212) = 11.94 to 23.32, all *p* < 0.001].

**Table 9:** Linear mixed-model estimates of the Study 4 intervention effects (54 dormitory clusters, 108 participants).

Outcome	T1 x group b (SE)	*z*	*p*	T2 x group *b* (SE)	*z*	*p*	*d* (T1)	*d* (T2)
Perceived cost	−0.56 (0.15)	−3.76	< 0.001	−0.51 (0.15)	−3.43	< 0.001	−0.88	−0.76
Compensation	0.71 (0.15)	4.61	< 0.001	0.66 (0.15)	4.29	< 0.001	1.07	0.95
Relational balance	0.75 (0.17)	4.44	< 0.001	0.70 (0.17)	4.14	< 0.001	1.10	0.98
Sustained willingness	0.64 (0.15)	4.30	< 0.001	0.61 (0.15)	4.10	< 0.001	0.95	0.87
Sense of community	0.68 (0.13)	5.23	< 0.001	0.63 (0.13)	4.85	< 0.001	1.06	0.94

Models used a dormitory-level random intercept (all five outcomes converged under this specification); dormitory ICCs ranged from 0.11 to 0.51. Intervention and control conditions did not differ at baseline on any outcome (all *p* > 0.75). Mixed repeated-measures ANOVA sensitivity analyses yielded the same pattern (time-by-group interaction F between 11.94 and 23.32, all *p* < 0.001). d values are between-condition standardized mean differences at each follow-up. A serial mediation model on T0-to-T1 change scores (5,000 dormitory-cluster bootstrap draws) supported the chain intervention -> compensation -> balance -> willingness [estimate = 0.042, 95% CI (0.003, 0.115)] and the balance pathway [estimate = 0.120, 95% CI (0.018, 0.270)], but not the compensation-only pathway [estimate = 0.061, 95% CI (-0.079, 0.229)]; the direct effect remained significant [estimate = 0.417, 95% CI (0.018, 0.791)], indicating partial mediation.

#### 5.5.2 Sequential indirect association

The dormitory-cluster bootstrap analysis supported the proposed sequence: the indirect path from condition to willingness change via balance change was significant [*b* = 0.12, 95% CI (0.02, 0.27)], as was the sequential path through compensation change and then balance change [*b* = 0.04, 95% CI (0.00, 0.11)]; the path through compensation change alone was not significant [*b* = 0.06, 95% CI (−0.08, 0.23)]. The direct effect of condition on willingness change remained significant [*b* = 0.42, 95% CI (0.02, 0.79)], indicating partial mediation in which compensation gains operate as a precursor that restores relational balance, which in turn supports willingness.

## 6 General discussion

### 6.1 Kindness beneath the eaves: the behavioral spectrum of implicit prosocial behavior

Across four studies, this project examined implicit prosocial behavior, informal, low-visibility, habitual helping, in the confined setting of university dormitories. The grounded analysis organized the behavior into daily assistance, emotional support, and spatial coordination, a three-dimensional spectrum covering life, emotion, and space. This spectrum extends prosocial research beyond its customary focus on overt, recordable acts (Penner et al., 2005) and connects it to emerging work on everyday kindness among university students (Cash et al., 2024): in dormitories, the consequential acts are precisely those that are not announced. That implicit helping was associated with balance, willingness, and community sense mirrors the broader prosociality-well-being link documented longitudinally and meta-analytically (Hui et al., 2020; Ju et al., 2025; Xiong et al., 2023; Yao et al., 2026), and fits the Chinese residential context in which dormitory interpersonal relations are central to student mental health (Sun, 2023).

### 6.2 Boundary costs: the most consequential cost in shared housing

Among the four costs, boundary cost carried the largest relative weight for sustained willingness. Dormitories are confined spaces with high interpersonal density; personal territory is already minimal, and persistent accommodation can further blur the boundaries of space, time, and privacy. This reading is consistent with privacy-regulation theory, which treats control over access to the self as a basic regulatory need (Altman, 1975), and with evidence that privacy maintenance is an ongoing negotiation in close relationships (Kil et al., 2022). The practical implication is that dormitory management should protect boundary needs alongside encouraging mutual support: helping students articulate and negotiate boundaries may prevent goodwill from turning into resentment.

### 6.3 From cost to balance: the balancing mechanism

Relational balance statistically accounted for about 62.0% of the concurrent cost-willingness association, and the intervention data showed that balance change transmits part of the program's effect on willingness change. This pattern supports the Study 1 account in which individuals do not respond to costs directly but to the perceived equilibrium between effort and reward, extending social exchange reasoning into the communal texture of dormitory life (Clark and Mills, 1979; Cropanzano and Mitchell, 2005). Because the survey-based decomposition is cross-sectional, we refrain from a temporal mediation claim (Maxwell and Cole, 2007); the convergence of the experimental compensation effects (Study 3) and the intervention sequence (Study 4), however, gives the balancing account triangulated support.

### 6.4 Implicit compensation: the stronger currency in shared living

Implicit rewards showed a significantly larger indirect association with balance than explicit rewards, and in the scenario experiment implicit rewards produced the highest sustained willingness at both cost levels. In a setting of frequent, close, long-term interaction, relationships are partly communal rather than purely transactional (Clark and Mills, 1979); what givers appear to seek is not only acknowledgment but evidence that the relationship itself is deepening. This aligns with gratitude research showing that appreciation works by strengthening relational bonds (Emmons and McCullough, 2003; Geier and Morris, 2022), and suggests that building felt closeness and being-neededness may be more effective for sustaining everyday helping than merely prompting reciprocal courtesy (Bohlmeijer et al., 2022; Weinstein and Ryan, 2010).

### 6.5 The buffering role of community and the intervention pathway

Dormitory sense of community moderated the cost-willingness association: at high community sense, the negative slope was clearly weaker, consistent with the idea that identification with the community reframes giving from personal loss to contribution to one's own group (Iwai and Tavares, 2024; McMillan and Chavis, 1986; Zarbo et al., 2025). The intervention translated this logic into practice: expression of costs, specific gratitude, reciprocal rituals, boundary negotiation, and community building produced gains that persisted at three months, and the cluster-bootstrap sequence indicated that compensation gains restored balance, which in turn sustained willingness. This expression-perception-normalization-respect-belonging pathway offers a workable template for residential advisors and counselors.

### 6.6 Limitations

Several limitations qualify these findings. First, Study 2 used only two waves; although the RI-CLPM separates trait from state variance, two-wave designs cannot fully identify within-person causal dynamics, and the cross-lagged estimates should be read as short-run directional influences pending replication with three or more waves (Hamaker et al., 2015; Mulder and Hamaker, 2021). Second, the survey-based indirect-effect decompositions are cross-sectional and can be biased relative to longitudinal mediation estimates (Maxwell and Cole, 2007); the reported percentages are descriptive. Third, all key measures are self-developed and self-reported; although the split-half psychometric evidence was encouraging, independent validation, ideally with informant reports or behavioral indicators, is needed. Fourth, Study 1 deliberately sampled frequent helpers, which may over-represent highly prosocial students and limit transferability to less engaged residents. Fifth, Study 3 used hypothetical scenarios, which supports internal but limits ecological validity, and the design was underpowered for the small interaction observed (achieved power approximately 0.16), so the descriptive high-cost amplification of implicit compensation requires confirmation. Sixth, in Study 4 only two members per dormitory enrolled, the number of clusters was modest (54), and outcomes were self-reported; whole-dormitory participation and objective behavioral outcomes would strengthen future trials. Finally, all samples came from Chinese universities; generalization to other residential and cultural contexts is an open question.

### 6.7 Conclusion

This project set out to describe the cost structure, compensation mechanisms, and community-building processes of implicit prosocial behavior in university dormitories. The evidence suggests that implicit helping in shared housing takes three forms and carries four costs, with boundary erosion weighing most heavily on students' willingness to continue. Cost perception predicted lower willingness over time within persons, while the reverse influence was negligible, and relational balance appeared to carry much of this association. Implicit relational rewards mattered more than explicit recognition for preserving balance, and a strong dormitory community softened the cost-willingness link. A reciprocity-based group program improved balance, willingness, and community sense, with gains maintained three months later, partly through restored balance. Taken together, the findings portray quiet everyday helping as a resource that is sustained not by selflessness alone but by balanced exchange, felt closeness, and community norms, and they point to concrete levers, recognizing unseen contributions, protecting boundaries, and cultivating reciprocal rituals, for building durable dormitory communities.

## Data Availability

The raw data supporting the conclusions of this article will be made available by the authors, without undue reservation.
