# Supplementary material for: Costs, compensations, and community building of implicit prosocial behavior in college dormitory environments: a mixed-methods study
Source: Front Psychol. 2026 Oct 19;17:1922584. doi: 10.3389/fpsyg.2026.1922584 (PMC13724609; doi:10.3389/fpsyg.2026.1922584)
Supplement: Supplementary file 1 [file Supplementary_File_1.docx]

**Supplementary Material**

*Costs, Compensations, and Community Building of Implicit Prosocial Behavior in College Dormitory Environments: A Mixed-Methods Study*

Jingwei Li, TianYu Li, Pengcheng Li

**Supplementary Table S1. Item pool of the six author-developed measures (5-point response scales; 1 = never or strongly disagree, 5 = always or strongly agree).**

| **Scale** | **Dimension** | **Item no.** | **Item wording** |
| --- | --- | --- | --- |
| Implicit Prosocial Behavior Scale | Daily assistance | IPB1 | I proactively take out the dormitory trash. |
| Implicit Prosocial Behavior Scale | Daily assistance | IPB2 | I refill the shared water dispenser without being asked. |
| Implicit Prosocial Behavior Scale | Daily assistance | IPB3 | I pick up parcels or meals for my roommates on my way back. |
| Implicit Prosocial Behavior Scale | Daily assistance | IPB4 | I lend my daily necessities to roommates when they need them. |
| Implicit Prosocial Behavior Scale | Daily assistance | IPB5 | I quietly tidy shared areas when they get messy. |
| Implicit Prosocial Behavior Scale | Emotional support | IPB6 | I listen to my roommates' worries. |
| Implicit Prosocial Behavior Scale | Emotional support | IPB7 | I notice when a roommate is upset and offer quiet company. |
| Implicit Prosocial Behavior Scale | Emotional support | IPB8 | I comfort my roommates when they fail exams or feel homesick. |
| Implicit Prosocial Behavior Scale | Emotional support | IPB9 | I keep the private matters my roommates share with me confidential. |
| Implicit Prosocial Behavior Scale | Emotional support | IPB10 | I mediate small frictions between roommates before they escalate. |
| Implicit Prosocial Behavior Scale | Spatial coordination | IPB11 | I adjust my routine to my roommates' schedules. |
| Implicit Prosocial Behavior Scale | Spatial coordination | IPB12 | I lower my voice or use earphones when roommates are resting or studying. |
| Implicit Prosocial Behavior Scale | Spatial coordination | IPB13 | I dim the lights or my screen when roommates are sleeping. |
| Implicit Prosocial Behavior Scale | Spatial coordination | IPB14 | I coordinate the use of shared space, such as desks and the balcony, without being asked. |
| Implicit Prosocial Behavior Scale | Spatial coordination | IPB15 | I move quietly when entering or leaving the dormitory at early or late hours. |
| Cost Perception Scale | Time cost | CP1 | Helping my roommates takes up much of my personal time. |
| Cost Perception Scale | Time cost | CP2 | My study schedule is often interrupted by roommates' needs. |
| Cost Perception Scale | Time cost | CP3 | I spend time on dormitory chores that I could use for myself. |
| Cost Perception Scale | Energy cost | CP4 | Looking after shared tasks leaves me physically tired. |
| Cost Perception Scale | Energy cost | CP5 | Frequent small favors drain my energy. |
| Cost Perception Scale | Energy cost | CP6 | I feel exhausted from constantly accommodating my roommates. |
| Cost Perception Scale | Emotional cost | CP7 | Suppressing my own preferences to keep the peace makes me feel wronged. |
| Cost Perception Scale | Emotional cost | CP8 | I feel emotionally drained when my efforts are taken for granted. |
| Cost Perception Scale | Emotional cost | CP9 | Caring for roommates' moods adds pressure to my own emotions. |
| Cost Perception Scale | Boundary cost | CP10 | My personal space feels eroded. |
| Cost Perception Scale | Boundary cost | CP11 | I have little privacy in the dormitory. |
| Cost Perception Scale | Boundary cost | CP12 | My belongings are used by roommates without my permission. |
| Cost Perception Scale | Boundary cost | CP13 | I cannot arrange my own corner of the room as I wish. |
| Compensation Perception Scale | Explicit rewards | COMP1 | My roommates express clear gratitude for what I do. |
| Compensation Perception Scale | Explicit rewards | COMP2 | My roommates return favors in concrete ways. |
| Compensation Perception Scale | Explicit rewards | COMP3 | My contributions are openly acknowledged in the dormitory. |
| Compensation Perception Scale | Explicit rewards | COMP4 | My roommates offer me tangible help in return. |
| Compensation Perception Scale | Implicit rewards | COMP5 | We are becoming closer. |
| Compensation Perception Scale | Implicit rewards | COMP6 | I feel that I am needed by my roommates. |
| Compensation Perception Scale | Implicit rewards | COMP7 | Our relationship grows warmer after I help. |
| Compensation Perception Scale | Implicit rewards | COMP8 | I sense unspoken appreciation from my roommates. |
| Relational Balance Scale | Relational balance | BAL1 | My effort and reward feel balanced. |
| Relational Balance Scale | Relational balance | BAL2 | I am satisfied with the sense of fairness in my dormitory relationships. |
| Relational Balance Scale | Relational balance | BAL3 | What I give to my roommates matches what I receive. |
| Relational Balance Scale | Relational balance | BAL4 | The give-and-take in our dormitory feels equitable. |
| Relational Balance Scale | Relational balance | BAL5 | My dormitory relationships feel mutually rewarding. |
| Relational Balance Scale | Relational balance | BAL6 | The balance between giving and receiving in our dormitory is stable over time. |
| Sustained Willingness Scale | Sustained willingness | W1 | I am willing to keep helping my roommates. |
| Sustained Willingness Scale | Sustained willingness | W2 | I am willing to maintain my current level of effort. |
| Sustained Willingness Scale | Sustained willingness | W3 | I intend to continue caring for my roommates in the coming semester. |
| Sustained Willingness Scale | Sustained willingness | W4 | I will keep doing quiet favors for my roommates in the future. |
| Sustained Willingness Scale | Sustained willingness | W5 | Even without recognition, I am willing to go on contributing to our dormitory. |
| Dormitory Sense of Community Scale | Belonging | COMM1 | I feel that I belong to this dormitory. |
| Dormitory Sense of Community Scale | Belonging | COMM2 | This dormitory feels like a small home to me. |
| Dormitory Sense of Community Scale | Belonging | COMM3 | I identify with our dormitory as a community. |
| Dormitory Sense of Community Scale | Trust | COMM4 | I can trust my roommates with personal matters. |
| Dormitory Sense of Community Scale | Trust | COMM5 | My roommates are reliable when I need help. |
| Dormitory Sense of Community Scale | Trust | COMM6 | I feel safe being myself in this dormitory. |
| Dormitory Sense of Community Scale | Reciprocity norms | COMM7 | In our dormitory, helping each other is the norm. |
| Dormitory Sense of Community Scale | Reciprocity norms | COMM8 | People in this dormitory return kindness with kindness. |
| Dormitory Sense of Community Scale | Reciprocity norms | COMM9 | We have shared expectations about looking out for one another. |

All items were generated from the grounded categories of Study 1 and reviewed by two subject-matter experts for content validity before administration.

**Supplementary Table S2. Standardized confirmatory factor loadings of the six measures (holdout half, n = 928).**

| **Scale** | **Item no.** | **Standardized loading** |
| --- | --- | --- |
| Implicit Prosocial Behavior Scale | IPB1 | 0.730 |
| Implicit Prosocial Behavior Scale | IPB2 | 0.727 |
| Implicit Prosocial Behavior Scale | IPB3 | 0.733 |
| Implicit Prosocial Behavior Scale | IPB4 | 0.664 |
| Implicit Prosocial Behavior Scale | IPB5 | 0.728 |
| Implicit Prosocial Behavior Scale | IPB6 | 0.703 |
| Implicit Prosocial Behavior Scale | IPB7 | 0.689 |
| Implicit Prosocial Behavior Scale | IPB8 | 0.709 |
| Implicit Prosocial Behavior Scale | IPB9 | 0.771 |
| Implicit Prosocial Behavior Scale | IPB10 | 0.688 |
| Implicit Prosocial Behavior Scale | IPB11 | 0.616 |
| Implicit Prosocial Behavior Scale | IPB12 | 0.707 |
| Implicit Prosocial Behavior Scale | IPB13 | 0.744 |
| Implicit Prosocial Behavior Scale | IPB14 | 0.686 |
| Implicit Prosocial Behavior Scale | IPB15 | 0.738 |
| Cost Perception Scale | CP1 | 0.855 |
| Cost Perception Scale | CP2 | 0.829 |
| Cost Perception Scale | CP3 | 0.834 |
| Cost Perception Scale | CP4 | 0.892 |
| Cost Perception Scale | CP5 | 0.888 |
| Cost Perception Scale | CP6 | 0.832 |
| Cost Perception Scale | CP7 | 0.859 |
| Cost Perception Scale | CP8 | 0.837 |
| Cost Perception Scale | CP9 | 0.888 |
| Cost Perception Scale | CP10 | 0.891 |
| Cost Perception Scale | CP11 | 0.838 |
| Cost Perception Scale | CP12 | 0.847 |
| Cost Perception Scale | CP13 | 0.836 |
| Compensation Perception Scale | COMP1 | 0.793 |
| Compensation Perception Scale | COMP2 | 0.767 |
| Compensation Perception Scale | COMP3 | 0.858 |
| Compensation Perception Scale | COMP4 | 0.742 |
| Compensation Perception Scale | COMP5 | 0.775 |
| Compensation Perception Scale | COMP6 | 0.773 |
| Compensation Perception Scale | COMP7 | 0.743 |
| Compensation Perception Scale | COMP8 | 0.802 |
| Relational Balance Scale | BAL1 | 0.715 |
| Relational Balance Scale | BAL2 | 0.728 |
| Relational Balance Scale | BAL3 | 0.673 |
| Relational Balance Scale | BAL4 | 0.662 |
| Relational Balance Scale | BAL5 | 0.717 |
| Relational Balance Scale | BAL6 | 0.753 |
| Sustained Willingness Scale | W1 | 0.762 |
| Sustained Willingness Scale | W2 | 0.836 |
| Sustained Willingness Scale | W3 | 0.763 |
| Sustained Willingness Scale | W4 | 0.776 |
| Sustained Willingness Scale | W5 | 0.776 |
| Dormitory Sense of Community Scale | COMM1 | 0.819 |
| Dormitory Sense of Community Scale | COMM2 | 0.797 |
| Dormitory Sense of Community Scale | COMM3 | 0.790 |
| Dormitory Sense of Community Scale | COMM4 | 0.820 |
| Dormitory Sense of Community Scale | COMM5 | 0.785 |
| Dormitory Sense of Community Scale | COMM6 | 0.769 |
| Dormitory Sense of Community Scale | COMM7 | 0.772 |
| Dormitory Sense of Community Scale | COMM8 | 0.832 |
| Dormitory Sense of Community Scale | COMM9 | 0.784 |

Loadings are from the confirmatory factor analysis in the holdout half of the Study 2 sample; all loadings were significant (p < 0.001). Item dimensions are as listed in Supplementary Table S1.
